# Mapping the Interactions between a RUN Domain from DENND5/Rab6IP1 and Sorting Nexin 1

**DOI:** 10.1371/journal.pone.0035637

**Published:** 2012-04-25

**Authors:** Humberto Fernandes, Edward Franklin, Florence Jollivet, Katharina Bliedtner, Amir R. Khan

**Affiliations:** School of Biochemistry and Immunology, Trinity College, Dublin, Ireland; BioScience Project, United States of America

## Abstract

Eukaryotic cells have developed a diverse repertoire of Rab GTPases to regulate vesicle trafficking pathways. Together with their effector proteins, Rabs mediate various aspects of vesicle formation, tethering, docking and fusion, but details of the biological roles elicited by effectors are largely unknown. Human Rab6 is involved in the trafficking of vesicles at the level of Golgi *via* interactions with numerous effector proteins. We have previously determined the crystal structure of Rab6 in complex with DENND5, alternatively called Rab6IP1, which comprises two RUN domains (RUN1 and RUN2) separated by a PLAT domain. The structure of Rab6/RUN1-PLAT (Rab6/R1P) revealed the molecular basis for Golgi recruitment of DENND5 *via* the RUN1 domain, but the functional role of the RUN2 domain has not been well characterized. Here we show that a soluble DENND5 construct encompassing the RUN2 domain binds to the N-terminal region of sorting nexin 1 by surface plasmon resonance analyses.

## Introduction

Eukaryotic cells rely on an intricate trafficking system to shuttle proteins, lipids, and other vesicular cargo between sub-cellular compartments. Trafficking involves protein/protein and protein/lipid interactions leading to vesicle formation, tethering, docking and fusion. These steps are regulated to provide specificity and preserve cellular structure and organelle identity, such as the Golgi apparatus, endosomes and lysosomes [1]–[3]. The specificity between donor and acceptor membranes is widely believed to be mediated by the Rab family of small GTPases, which comprise the largest member of the Ras superfamily [4]. Active Rabs are non-covalently associated with GTP and localize to distinct sub-cellular compartments *via* prenylated C-terminal tails [5], [6]. Recruitment of effector proteins, which recognize the active conformation, leads to the regulation of various steps in vesicle delivery. Inactivation of Rabs follows hydrolysis of GTP, aided by GTPase activating proteins (GAPs), and Rabs are subsequently extracted from the membrane into the cytosolic fraction by GDP dissociation inhibitor [7].

Rab6 regulates anterograde and retrograde traffic at the level of Golgi *via* interactions with numerous and unrelated effector proteins [8], [9]. Rab6A and Rab6A’ are ubiquitously expressed in cells, whereas expression of a third isoform – Rab6B – is restricted to brain tissue [10]. A fourth isoform, Rab6C, has recently been shown to encode a brain-specific retrogene with an unusual GTP-binding motif that localizes to the centrosome and regulates cell cycle progression [11]. One of the most widely studied effectors of Rab6 is DENND5/Rab6IP1 protein (Differentially Expressed in Neoplastic versus Normal Cell; alternatively called Rab6-Interacting Protein 1) [12]. DENND5 was initially identified by yeast two-hybrid assays as an effector of Rab6 [13]. The N-terminal half of the 1287-residue DENND5A isoform is composed of a series of the eponymous DENN domains that appear to function as a GDP/GTP exchange factor (GEF) for Rab39 [14]. The C-terminal half of the effector is composed of two RUN domains (RUN1 and RUN2) separated by a PLAT domain. In previous work, we determined the crystal structure of Rab6A in complex with the tandem RUN1-PLAT domains of DENND5A [15]. The structure revealed the molecular basis for Rab6-mediated recruitment of DENND5 to Golgi, as well as the orientation of the lipophilic loops of the PLAT domain, relative to the Rab-binding interface.

Despite numerous cellular and structural studies of Rab6 effectors, the role of the RUN2 domain of DENND5 remains unknown. RUN domains, named after RPIP8 (Rap2 interacting protein 8), UNC-14 and NESCA (new molecule containing SH3 at the carboxyl-terminus) [16], are widely occurring modules that appear to have diverse roles in cell signaling [17]. Rap2-Interacting Protein X (RPIPX) also contains a RUN domain and belongs to a family of effectors that bind to the small GTPase Rap2 [18], [19]. The uncomplexed crystal structure of the RUN domain of RPIPX has been determined [20]. However, RUN domains appear to have roles beyond small GTPase signaling [21]. Recently, an interaction between DENND5 and sorting nexin 1 (SNX1) has been reported by a genome-wide yeast two-hybrid screen and GST pulldowns [22]. SNX1 forms a transient complex with the retromer in mammalian cells to drive vesicle transport between early endosomes and the trans-Golgi network [23]–[25]. Human SNX1 consists of an N-terminal sorting nexin (SNX) region, a central PX (Phox-homology) domain, and a C-terminally situated BAR domain (Bin, amphiphysin and Rvs) that binds to and/or induces membrane curvature *via* interactions with the lipid bilayer [26]–[28]. Here we report that the RUN2 domain of DENND5 binds to the N-terminal SNX region of SNX1 by surface plasmon resonance analyses.

## Methods

### DENND5 Expression and Purification

Cloning, expression and purification of Rab6 and an engineered RUN1-PLAT (RPdel) construct has been published previously [29]. In brief, a truncated version of human Rab6a encompassing residues 8 to 195 (Q72L mutant), with a thrombin cleavable N-terminal His-tag was used in this study. An engineered variant of mouse DENND5A containing a loop deletion between residues 813–835 (inclusive) of RUN1, henceforth referred to as RPdel, was also used [29]. RPdel denotes the RUN-PLAT tandem domains of DENND5A. This protein was expressed as an N-terminal His-tagged protein containing a tobacco etch virus (TEV) cleavable N-terminal His-tag. Both proteins were expressed in *E. coli* individually or co-expressed and purified to homogeneity.

Cloning, expression and purification of the RUN1-PLAT-RUN2 (RPRdel; residues 716–1278) region of DENND5A, was performed using similar strategies [29], with the exception of the reverse primer (**Table 1**). Cloning of the fragment was derived from a synthetic gene comprising the entire coding region of DENND5 (Geneart AG), with or without the cDNA corresponding to the loop 813–835 of RUN1. However, both the wild-type RPR protein and RPRdel were insoluble when expressed alone in *E. coli*. Co-expression with Rab6a increased the solubility of the three-domain constructs, therefore the complex Rab6a/RPRdel was purified as outlined in previous work and used for binding studies.

**Table 1:** Oligonucleotide primers used for cloning of DENND5 and SNX1 variants.

Protein	Variant (residue range)	Primers (5′→3′)	
DENND5	RPdel (702–1057)	N	CAGGATCCATGGGCAGTACCATCCGTG
		C	CGGAATTCTCAGGACTGCTGTAGTGGCGGAGT
	RPRdel (702–1287)	N	CAGGATCCATGGGCAGTACCATCCGTG
		C	TATCCACCTTTACTGTTAAATATCAATGCCTTTAACCAGGCTGGT
SNX1	SNX-PX (9–303)	N	TACTTCCAATCCATGAGCGCAAGCGAACGTCTGCCTCCG
		C	TATCCACCTTTACTGTTATTCATTCATTTTAATGGTCATTTGC
	PX-BAR (141–521)	N	TACTTCCAATCCATGGATCAGTTTGATCTGACCGTTGG
		C	TATCCACCTTTACTGTTAGCTAATGGCTTTTGCTTCCGGCAG
	PX (141–303)	N	TACTTCCAATCCATGGATCAGTTTGATCTGACCGTTGG
		C	TATCCACCTTTACTGTTATTCATTCATTTTAATGGTCATTTGC
	BAR (300–521)	N	TACTTCCAATCCATGAATGAAAGCGATATTTGGTTTG
		C	TATCCACCTTTACTGTTAGCTAATGGCTTTTGCTTCCGGCAG

### SNX1 expression and purification

**Figure 1:**
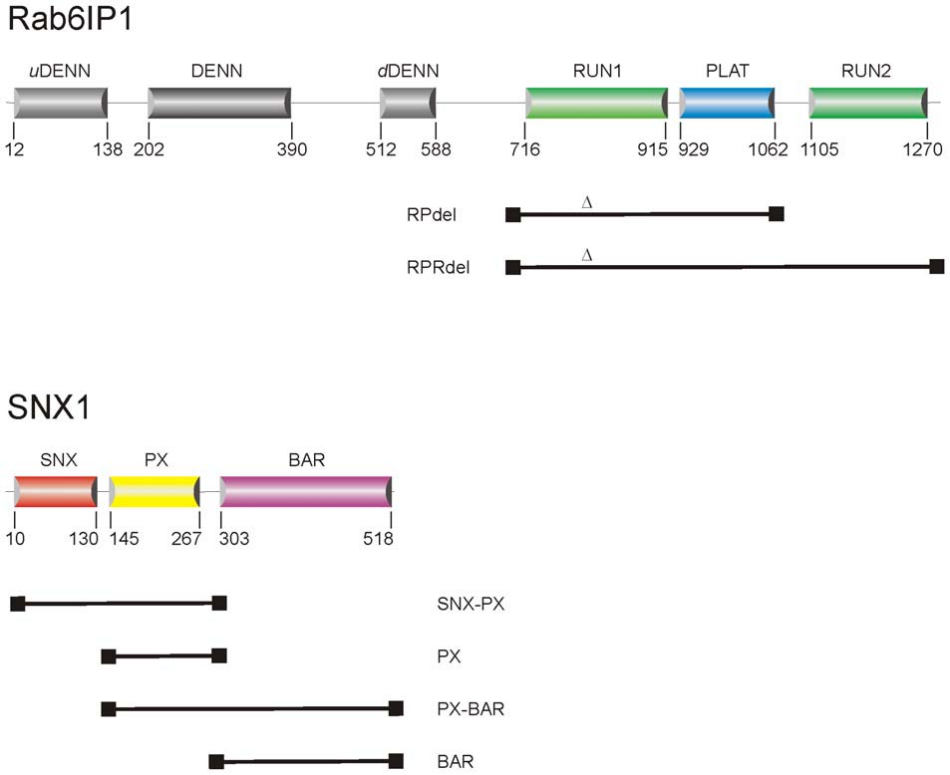
The domain organization of DENND5 and SNX1. Constructs used in the mapping studies are shown below the domains. The engineered loop deletion in DENND5 between α3 and α4, denoted by Δ (residues 813–835), rendered the protein soluble and facilitated its purification.

A near full-length version (residues 9–521) of the mouse SNX1 gene (Gene ID 56440) was generated by Geneart AG (Regensburg, Germany). Four truncated constructs spanning different parts of the protein were sub-cloned from the parent Geneart construct (**Figure 1**; **Table 1**). The PCR fragments were ligated into the expression vector pNIC28-BSA4 which was linearized with the endonuclease BsaI [GenBank Accession No. EF198106; [30]], which encodes an N-terminal fusion peptide (MHHHHHHSSGVDLGTENLYFQ*SM) that encompasses a His-tag and an rTEV cleavage site (*).

The various constructs were transformed into *E. coli* BL21 (DE3) cells and all subsequent cultures were grown in 2xYT medium supplemented with 34 mg/L kanamycin. For large-scale cultures, 1 litre 2xYT was inoculated with 10 ml of an overnight culture. The cells were harvested by centrifugation at 2700×*g* for 10 minutes. The bacterial pellets were washed once with ice-cold phosphate-buffered saline buffer and stored as frozen pellets at 253 K.

For purification, the pellets were resuspended in 300 mM NaCl, 5 mM MgCl_2_, 10 mM imidazole, 20 mM β-mercaptoethanol, 0.5 mM PMSF, and 10 mM Tris-HCl (pH 8.0). The cells were disrupted by sonication and the lysates centrifuged at 20000×*g* for 60 minutes to eliminate cellular debris. The cleared lysate was decanted, passed through a 0.2 µm filter, and loaded onto a gravity column containing 2 mL of Ni^2+^-agarose ChroMatrix (Jena Biosciences). The slurry was washed with ten column volumes of 300 mM NaCl, 5 mM MgCl_2_, 10 mM imidazole, 20 mM β-mercaptoethanol, 10 mM Tris-HCl (pH 8.0), followed by five column volumes of the same buffer but with increased imidazole (40 mM). Finally, the proteins were eluted with a step gradient to 200 mM imidazole, along with the various components of the wash buffer. The hexahistidine tag was cleaved by overnight dialysis in 150 mM NaCl, 5 mM MgCl_2_, 20 mM β-mercaptoethanol, 10 mM Tris-HCl (pH 8.0) using 10 µg of rTEV per milligram of eluted protein. Following dialysis, the proteins were supplemented with NaCl and imidazole to final concentrations of 300 mM and 10 mM, respectively. The rTEV protease, the cleaved tag and remaining uncleaved SNX1 protein remained bound to the resin during a second passage through a Ni^2+^-agarose ChroMatrix column, while the cleaved SNX1 protein was collected as the flow-through sample.

Affinity-purified proteins were then dialyzed at 277 K for three hours against 40 mM NaCl, 5 mM DTT, 10 mM Tris-HCl (pH 8.0). After dialyzing, the proteins were loaded onto a MonoQ anion exchange column (GE Healthcare) and eluted with a NaCl linear gradient from 40–500 mM. Fractions containing the SNX1 proteins were pooled, concentrated, and loaded onto a Superdex-200 size-exclusion column (GE Healthcare) equilibrated with 100 mM NaCl, 5 mM DTT, 10 mM Tris-HCl (pH 8.0).

### Cellular localization by confocal microscopy

The full-length human DENND5A gene (Gene ID 19347), lacking the cDNA corresponding to residues 813-LSTSGILLDSERRKSDASAVMSP-835, was synthesized by Geneart AG (Regensburg, Germany). The synthetic gene was sub-cloned into the pEGFP-C3 vector at the SacI/EcoRI site. HeLa cells were grown in DMEM containing 4.5 g/l glucose supplemented with 10% fetal calf serum, penicillin-streptomycin, and sodium pyruvate in a 5% humidified CO_2_ incubator. Transfection was achieved using the calcium phosphate precipitate method described [31]. HeLa Cells were transfected for 48 hours and processed for immunofluorescence analysis of the recombinant protein, henceforth referred to as DENND5del, as previously described [32]. Control experiments involved the full-length wild-type protein DENND5A transfected into HeLa cells under identical conditions. Green fluorescent protein (GFP) labeled DENND5A (WT and deletion variants) were analyzed for co-localization with full-length CherryFP-labeled Rab6A upon co-transfection of the two constructs. Alexa Fluor–labeled secondary antibodies were obtained from Molecular Probes (Eugene, OR), and Cy3 labeled secondary antibody was obtained from Jackson ImmunoResearch Laboratories (West Grove, PA). Anti-GM130 was from BD Biosciences (San Jose, CA) and was stained using Cy3 labeled secondary antibody. Alternatively the natural fluorescence of GFP, YFP and mCherry was used. The optical microscope used was a Leica DMRA (Wetzlar, Germany) equipped with a Micromax cooled CCD camera controlled by the Metamorph software (Molecular Devices, Berkshire, United Kingdom). Images were acquired using the Leica 63× numerical aperture (NA) 1.32, with len immersed in oil, contrast mode ph3, objective lens type HCX PL APO, and fluorescence filters A4, L5, Y3, and Y5.

### Surface Plasmon resonance

A Biacore X-100 instrument (GE healthcare) was used for collection of surface plasmon resonance (SPR) data, and all binding experiments were carried out at 293 K. The complexes Rab6/RPRdel or Rab6/RPdel were coupled as ligands to a CM5 chip using standard amine coupling procedure *via* 1-(3-dimethylaminopropyl)-3-ethylcarbodiimide hydrochloride and *N*-hydroxysuccinimide to a level of approximately 2,500 Resonance Units (RU) on flow cell 2. Although a non-covalent complex was initially coupled, it is very likely that one of the two proteins dissociated following a wash of the derivatized chip. Since RPRdel (60 kDa) and RPdel (40 kDa) are larger than Rab6 (20 kDa), it was expected that significant amounts of the effector were actively coupled to the CM5 chip, and also for this reason, a high density of protein was coupled to the chip.

SPR data were subtracted from the non-derivatized surface (flow cell 1) in all subsequent binding analyses. In order to minimize buffer mis-match, the analytes (SNX1 fragments) were dialysed against running buffer (100 mM NaCl, 1 mM DTT, 0.005% P20, 10 mM HEPES pH 8.0) before injection. The experiments were performed in multi-cycle mode with regeneration of the chip following each injection/dissociation step. The regeneration condition was optimized and depending on the experimental conditions and ligand coupled to the chip, a solution of 10 mM glycine (pH 2.5) or 4 M NaCl was utilized for stripping the analyte without significant loss of activity on the chip. Various concentrations of analyte (SNX1 fragments) ranging from 0.2 to 40 µM were used in the binding experiments. One of the concentrations, typically 1 µM, was duplicated as an internal control. A buffer duplication (0 µM concentration of protein) was also performed during the series of injections. Prior to data processing, each set of injections were subtracted from the background (0 µM injection). The equilibrium dissociation constant (K_D_) was derived by fitting the data to a 1∶1 binding model using Biacore Evaluation software version 2.0.

## Results and Discussion

### Protein expression and purification

**Figure 2:**
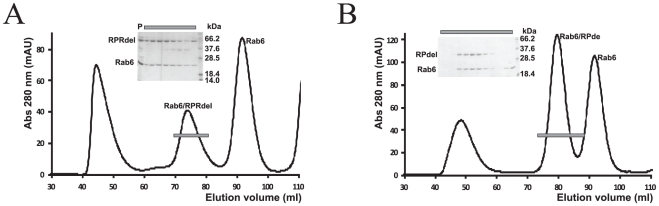
Purification of Rab6/DENND5 complexes by gel filtration chromatography. (A) Elution of Rab6/RPRdel. (B) elution of Rab6/RPdel. The bars denote the samples analyzed by SDS-PAGE (inset).

The RUN2 domain of DENND5 was successfully expressed and purified to homogeneity (data not shown). However, upon coupling of the isolated DENND5 domain to CM5 chips, no significant interactions could be detected with various constructs of SNX1. Alternatively, various constructs of SNX1 could not be coupled actively to CM5 sensor chips, possibly due to the elongated structure and chemistry of the PX and BAR domains. These technical obstacles were overcome by soluble expression of the complete C-terminal half of DENND5 (residues 702–1287), which comprises the RUN1-PLAT-RUN2 domains (RPRdel; **Figure 2**). The RPRdel construct could not be expressed solubly alone, and therefore, it was necessary to co-express with Rab6 to obtain a soluble non-covalent complex (Rab6/RPRdel) in milligram amounts for binding studies. During the derivatization of CM5 chips, it is probable that only one of the two proteins, Rab6 or RPRdel, was coupled to chips, while the non-covalently associated partner would be expected to dissociate. Since RPRdel (60 kDa) and RPdel (40 kDa) are much larger than Rab6, we feel that derivatization of the CM5 chip would be dominated by the effector. In addition, a high density of protein was coupled to the surface (2,500 RU), so the chip is expected to have significantly active amounts of the effector.

**Figure 3:**
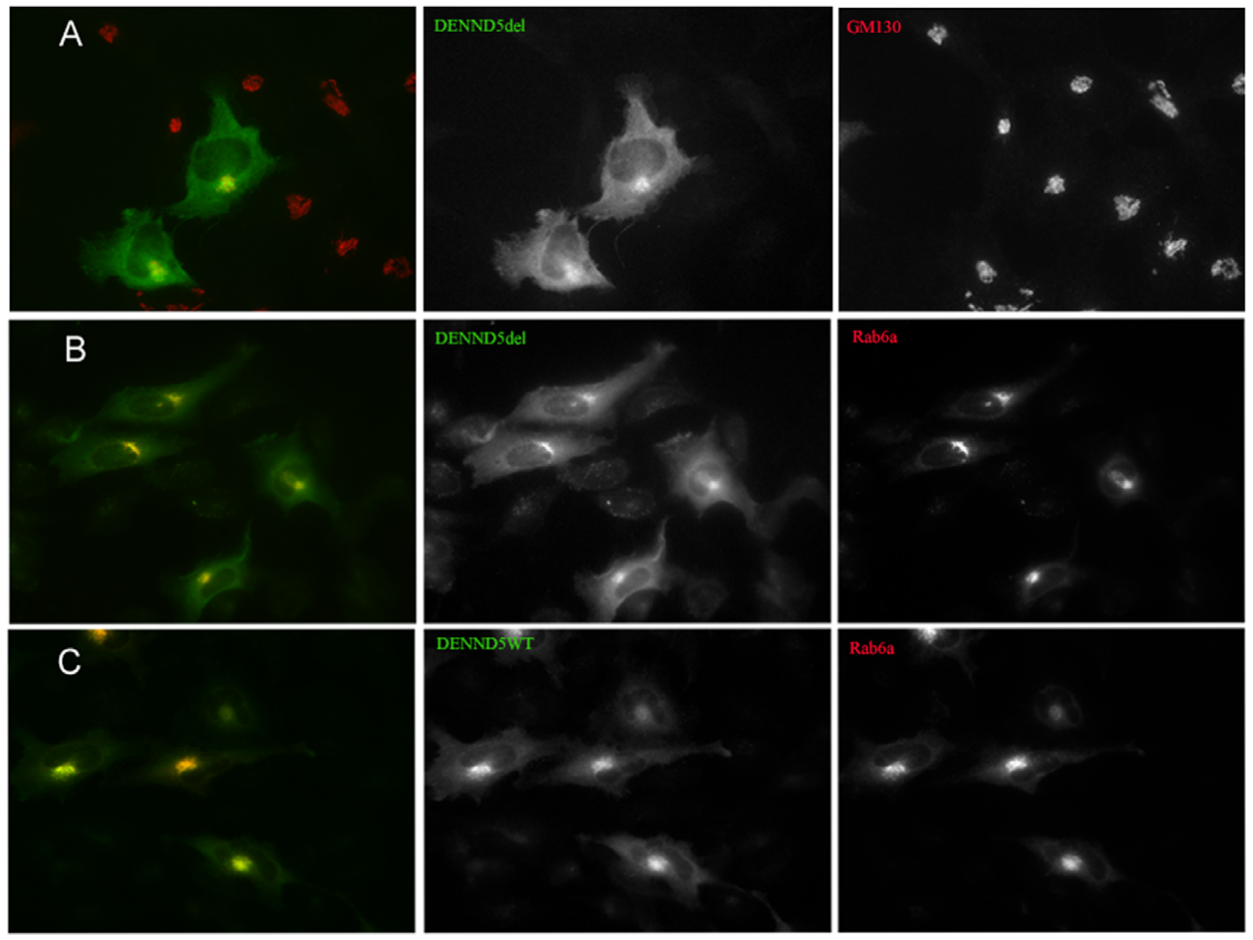
Immune co-localization studies of Rab6 and DENND5. (A) transfection and overlay with GFP-DENND5del (green) and GM130 (Cy3; red). The black and white panels are the same image without colour, revealing the expression of each protein in the transfected cells. (B) Transfection with GFP-DENND5del and wild-type Rab6A (mCherry; red), with the overlap apparent from the yellow colour. (C) Transfection with YFP-DENND5 (wild-type) and mCherry-Rab6A (wild-type), revealing co-localization in Golgi compartments.

The loop deletion in RUN1 (RPRdel) enhanced its solubility upon co-expression with Rab6 in *E.coli*, and did not affect the recruitment of the full-length protein to Golgi. Overlays of the immunofluorescence of GFP-labelled wild-type DENND5del and Golgi-resident GM130 (**Figure 3A**) reveal the Golgi localization of the effector containing the engineered loop deletion. The row of panels in **Figure 3B** is the co-expression of the full-length variant, DENND5del, with Rab6a (red) showing that the proteins co-localize. In order to highlight the co-localization, the non-overlayed cell expression is duplicated in adjacent gray scale images. Although there is an excess of green background in these panels, due to high levels of effector expression, there is nevertheless co-localization. The last row of panels (**Figure 3C**) is a positive control showing the co-localization of wild-type DENND5 and Rab6-labelled Golgi membranes.

**Figure 4:**
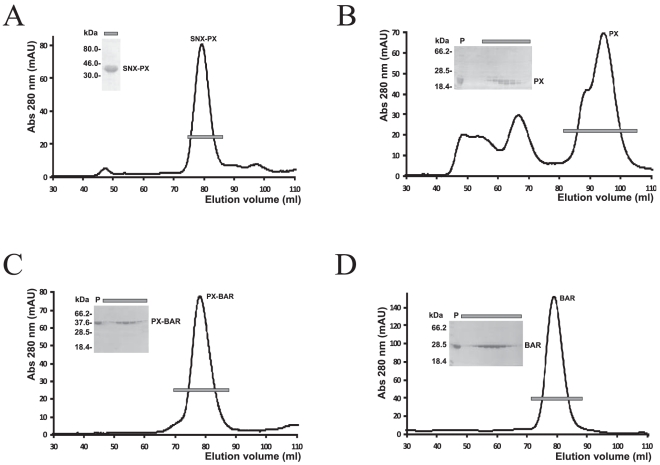
Purification of various SNX1 fragments by gel filtration chromatography. Lanes from SDS-PAGE gels (inset) correspond to the bars across the peaks.

As a control for the SPR experiments, the complex Rab6/RPdel was also purified to homogeneity (**Figure 2B**) and coupled to a CM5 chip. This construct lacked the C-terminal RUN2 domain and therefore provided an ideal negative control for binding to SNX1 fragments. Despite the non-covalent nature of the Rab6/effector complexes prior to covalent coupling onto sensor chips, there was no significant loss in sensor activity during buffer washes and SNX1 binding studies. We successfully purified numerous variants of SNX1 in milligram amounts and performed surface plasmon resonance analyses of their interactions with DENND5. The binding analyses were performed in parallel, on two separate CM5 chips coupled with DENND5del in the presence (RPRdel) or absence (RPdel) of the C-terminal RUN2 domain (**Figure 4**). These SNX1 variants were used to identify the segments that recognize DENND5. Most protein fragments were homogenous, although the PX domain of SNX1 was always purified as a doublet band, despite the presence of protease inhibitors during extraction and a rigorous purification regime (**Figure 4B**). Both bands migrated faster in SDS-PAGE gels upon cleavage by rTEV (not shown). This may indicate partial proteolysis at the C-terminal end of the protein, since the PX domain was expressed with a hexahistidine tag and rTEV site at the N-terminus.

### Mapping of the RUN2-interacting region of SNX1

**Figure 5:**
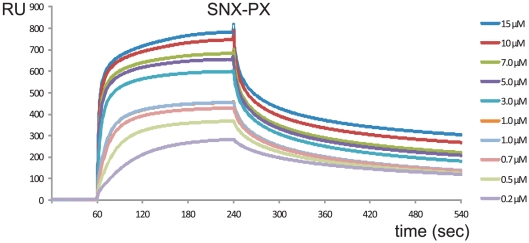
Surface plasmon resonance analyses of SNX1 binding to DENND5. The SNX-PX protein fragment was injected onto a Rab6/RPRdel coupled CM5 chip at various concentrations ranging from 0 to 15 µM.

**Figure 6:**
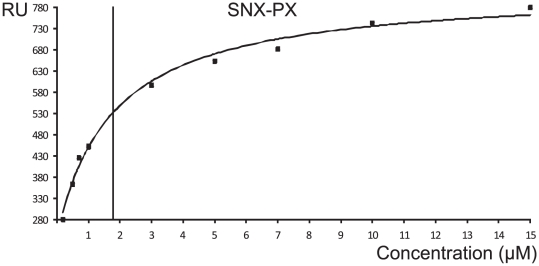
Fitting of SPR data using Biacore evaluation software. The end-points of the various injections were plotted against protein concentration and the hyperbolic curve was fit to a 1∶1 binding model. The vertical line represents the estimate of K_D_ at half-maximal binding of the ligand and analyte.

**Figure 7:**
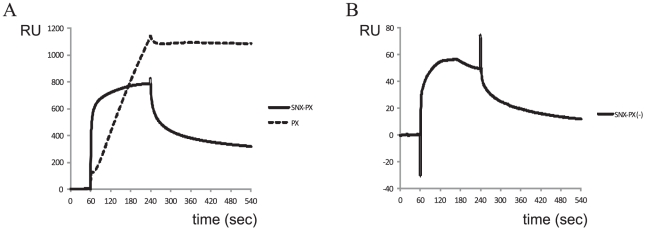
Control SPR experiments to map the binding segment of SNX1. (A) Injection of equivalent 15 µM amounts of SNX-PX and PX domains are superimposed. A CM5 chip coupled with RPRdel was used in these experiments. (B) Injection of 30 µM SNX-PX protein onto a CM5 chip coupled with Rab6/RPdel. Note that the magnitude of the binding, as evidenced by the *y*-axis (RU), is 10-fold smaller. These representative control experiments show that SNX-PX binds stably to DENND5-coupled chips that include the RUN2 domain. Data from the control experiments could not be processed and fit to conventional kinetics or equilibrium binding models using the evaluation software.

Purified SNX1 fragments were analyzed for binding to RPRdel using surface plasmon resonance experiments. Identical flow rates and association/dissociation parameters were utilized for all experiments. Ideal binding and dissociation curves were obtained upon injection of SNX-PX onto CM5 chips coupled with Rab6/RPRdel (**Figure 5**). The sensorgrams at various concentrations revealed conventional binding kinetics and were fit to a 1∶1 model (**Figure 6**), with an equilibrium dissociation constant (K_D_) of 1.7 µM. In contrast, the PX domain by itself revealed linear, slow, and non-saturable binding to the identical derivatized chip (**Figure 7A**). The sensorgram is indicative of non-specific aggregation and may indicate instability or unfolding of the PX domain and increased deposition onto the protein-coupled flow cell 2, relative to flow cell 1 (non-derivatized).

The data suggested that the SNX domain binds to Rab6/RPRdel coupled to the sensor surface of CM5 chips. As a control, purified Rab6/RPdel, lacking the RUN2 domain, was attached to a CM5 chip at a similar density. The binding properties of various SNX1 constructs were analyzed using identical injection parameters. In contrast to the Rab6/RPRdel complex, SNX-PX binding to Rab6/RPdel was 10-fold less (response units, RU) at twice the concentration (30 µM, **Figure 7B**) relative to binding of Rab6/RPRdel (15 µM, **Figure 7A**). In addition, the sensorgram revealed that the analyte SNX-PX was dissociating from the chip (**Figure 7B**) prior to completion of the 240 second injection pulse, suggesting an unstable complex. Therefore, the data are consistent with the stable binding of the N-terminal SNX region to the RUN2 domain of DENND5.

In summary, the only binary interaction that could be fit successfully to a conventional 1∶1 binding model was the complex between the tandem SNX-PX region and the coupled protein complex, Rab6/RPRdel. The affinity is relatively weak (K_D_ = 1.7 µM), but the low micromolar binding affinity is similar to the strength of Rab6 interactions with numerous effectors [9]. It cannot be conclusively ruled out, however, that the PX and BAR domains also contribute to the binding of RUN2. Recently, a relatively low affinity (1.6 µM) interaction between FIP5 (a Rab11 effector) and SNX18 has been identified, which contributes to endosomal trafficking in epithelial cells [33]. Interestingly, similar to our findings, the authors identified a low complexity (LC) region of SNX18, which lies ahead of the PX and BAR domains, as the binding site for FIP5. Previously, the weak affinities of Rab6 with a variety of unrelated effector proteins, along with rapid on/off kinetics, has been linked to the trafficking role of Rab6 in mediating transient tethering interactions [9]. The closely related SNX2 protein, which shares only 25% sequence identities with SNX1 in the SNX region (72% with the PX-BAR domains), is unable to bind DENND5 [22]. These observations are consistent with the notion that the SNX region encodes specificity. DENND5 also does not interact with SNX4, a sorting nexin protein with a similar domain organization, as evidenced by GST pulldowns [22]. Indeed, the SNX and PX regions of SNX1 mediate binding to Vps35 and Vps29, whereas SNX2 does not bind to the core retromer subunits [34]. Our mapping studies suggest a functional division of labour in which the N-terminus of SNX1 mediates vesicle specificity through interactions with Rab6/DENND5, and lipid attachment *via* the C-terminal PX-BAR domains. There are few studies describing the affinities or kinetics of protein/protein interactions that enable retromer-associated sorting pathways. The best example is the association of paralogues Vps26A and Vps26B with the heterodimer of Vps35/Vps29, which revealed an affinity (K_d_) of 8 nM and 4 nM, respectively, by isothermal titration calorimetry [35]. Thus, the interactions that are involved in formation of the core retromer are over two orders of magnitude stronger than the interaction between SNX1 and DENND5.

### Regulation of endosome/Golgi trafficking

In addition to Rab6 binding, DENND5 has previously been linked to the regulation of endosome/Golgi trafficking *via* its interactions with endosome-resident Rab11 [32]. The structure of Rab6 in complex with the RUN1-PLAT domains of DENND5 revealed that the recruitment of the effector is mediated by an all α-helical RUN1 domain [15]. The PLAT domain, which adopts a beta-sandwich fold, was rigidly associated with RUN1 *via* an intervening α-helix. Three loops connecting the beta-strands of the PLAT domain, enriched in positively-charged and aromatic residues, were oriented on the opposite end from the Rab6/RUN1 interface. PLAT domains have previously been observed to interact weakly with phospholipids [36]–[38]. Our data here suggest that the RUN2 domain associates with the N-terminal segment of SNX1 and potentially enables lipid binding *via* the PX and BAR domains. Several circumstantial pieces of evidence suggest that RUN2 is loosely associated with the rigid RUN1-PLAT tandem module. The C-terminus of the PLAT domain, preceding the RUN2 domain of DENND5, is flexible and the last 13 residues (1049–1061) are not seen in electron density maps of the Rab6/RUN1-PLAT crystal structure [15]. Also, incubation of purified RUN2 with the complex Rab6/RUN1-PLAT, followed by gel filtration chromatography, results in the elution of RUN2 and Rab6/RUN1-PLAT as separate peaks (data not shown).

**Figure 8:**
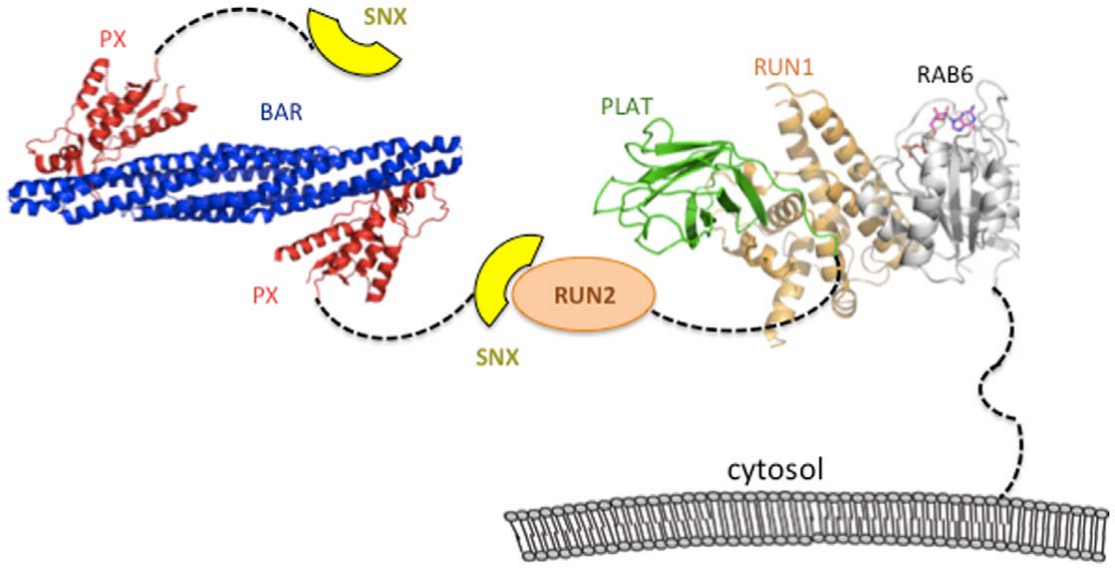
Model of the interactions between DENND5 and SNX1. Rab6 (grey) is tethered to Golgi *via* a flexible C-terminal segment that is prenylated at terminal cysteine residues. The two RUN domains (orange) are separated by a PLAT domain, whose distal loops may also interact with phospholipids. The RUN2 domain is shown interacting with the N-terminal SNX region (yellow) of a sorting nexin. The PDB code for the Rab6/DENND5 complex is 3cwz, and the structure of SNX9 (3rai) was used as a model for the PX-BAR domains of SNX1.

A model of the possible orientation of molecules is shown (**Figure 8**), using the PDB co-ordinates Rab6/DENND5 (3cwz) and the structure of the PX-BAR domains of the related molecule, SNX9 (3rai). The C-terminal tail (last 36 residues) of GTP-bound Rab6 is tethered to phospholipids *via* a prenylation site, and the RUN1-PLAT tandem region potentially interacts with the same or opposing bilayer [15]. The SNX region of SNX1 is predicted to be largely flexible and non-globular. For this reason, expression and purification of this segment in milligram amounts has proven elusive (data not shown). However, the PX-BAR domains are a rigid module that homo- and hetero-dimerize *via* the BAR domains [39], [40]. The banana-shaped BAR domain forms a positively-charged concave surface that promotes membrane curvature, while the PX domain binds to phosphatidyl-inositides such as phosphatidylinositol 3-monophosphate (PtdIns3P) [39], [41]. Deletion of the PX domain or truncation of the BAR domain disrupted vesicle trafficking from the endosome to the trans-Golgi network (TGN), indicating that both domains are required for proper cellular/membrane localization [28]. These structural properties of the PX-BAR tandem domains place steric and topological constraints on the multi-protein complex. The presence of unstructured links such as the C-terminal tail of Rab6, the segment linking PLAT and RUN2 domains, and the non-globular nature of the SNX region may provide the necessary flexibility to satisfy topological and steric constraints in complex formation.

The model presented in **Figure 8** is a simplification, since there is experimental evidence supporting dimerization of full-length DENND5 [32]. Thus, the dimerization capacities of both SNX1 and DENND5 may enable the formation of an oligomeric platform for lipid binding. In addition, it is not evident why RUN2 alone (coupled to CM5 chips) is unable to bind SNX1, although it may relate to better steric accessibility of RUN2 as part of the larger RUN1-PLAT-RUN2 fragment of DENND5. Furthermore, it is important to clarify that there is no evidence, either presented here or published previously, that directly supports the presence of a ternary complex of Rab6/DENND5/SNX1 *in vivo*. It is possible that Rab6/DENND5 and DENND5/SNX1 are binary and mutually exclusive complexes. However, there is circumstantial evidence for ternary complexes, given that TGN localization of DENND5 is dependent on Rab6. A fraction of endosomes carrying SNX1 were co-localized with TGN-associated DENND5, and suppression of DENND5 by siRNA resulted in loss of TGN-proximal SNX1-decorated endosomes [22]. Further structural and cellular studies are necessary for understanding the mechanism by which DENND5 regulates Golgi traffic *via* interactions with Rab6 and SNX1.
